# Assessing the effects of inulin‐type fructan intake on body weight, blood glucose, and lipid profile: A systematic review and meta‐analysis of randomized controlled trials

**DOI:** 10.1002/fsn3.2403

**Published:** 2021-06-21

**Authors:** Liangkui Li, Peng Li, Li Xu

**Affiliations:** ^1^ State Key Laboratory of Membrane Biology and Tsinghua‐Peking Center for Life Sciences School of Life Sciences Tsinghua University Beijing China

**Keywords:** blood glucose, diabetes, human subjects, inulin‐type fructans, meta‐analysis, total cholesterol

## Abstract

Inulin‐type fructan (ITF) intake has been suggested to alleviate several features of metabolic syndrome including obesity, diabetes, and hyperlipidemia; yet, results from the human trials remained inconsistent. We aimed to systematically evaluate the effects of ITF intake on body weight, glucose homeostasis, and lipid profile on human subjects with different health status, including healthy, overweight and obese, prediabetes and diabetes, and hyperlipidemia. Weighted mean differences (WMDs) between ITF and control groups were calculated by a random‐effects model. A total of 33 randomized controlled human trials were included. Significant effect of ITF intake was only observed in the diabetics, but not in the other subject groups. Specifically, ITF intervention significantly decreased the WMD of blood glucose (−0.42 mmol/L; 95% CI: −0.71, −0.14; *p* = .004), total cholesterol (−0.46 mmol/L; 95% CI: −0.75, −0.17; *p* = .002), and triglycerides (TAG) (−0.21 mmol/L; 95% CI: −0.37, −0.05; *p* = .01) compared with the control. The stability of these favorable effects of ITF on diabetics was confirmed by sensitivity analysis. Also, ITF tends to lower LDL cholesterol (*p* = .084). But body weight and blood insulin were not affected by ITF intake. It should be noted that blood glucose, total cholesterol, and LDL cholesterol exhibited high unexplained heterogeneity. In conclusion, ITF intake lowers blood glucose, total cholesterol, and TAG in the people with diabetes, and they may benefit from addition of inulin into their diets, but the underlying mechanisms responsible for these effects are inconclusive.

## Graphical abstract

Significant effect of inulin‐type fructans intake was only observed in the diabetics, but not in the other subject groups, inculding including healthy, overweight and obese, and hyperlipidemia.

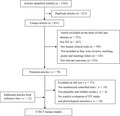

## 1 INTRODUCTION

In the last two decades, worldwide prevalence of lifestyle‐related chronic diseases such as obesity, diabetes, and cardiovascular diseases has risen to an epidemic level (Afshin et al., 2017; Wang et al., 2016; Wild et al., 2004). Epidemiological studies have consistently shown that diet rich in prebiotics reduces the risk of these lifestyle‐related diseases that plague the modern society (Pedersen et al., 2016; Shoaib et al., 2016; Slavin, 2013). Inulin‐type fructans (ITFs) are especially well known for their prebiotic effects (Hijova et al., 2013; Micka et al., 2017; Ning et al., 2017; Pool‐Zobel, 2005; Rault‐Nania et al., 2008; Shoaib et al., 2016; Yang et al., 2018).

ITF is a group of naturally occurring polysaccharides found in many plants (Roberfroid, 2005). They are classified according to their degree of polymerization (DP) into long‐chain inulin (chain length of 11–60 DP with an average of 25 DP) and fructo‐oligosaccharides (FOS) (chain length of 2–10 DP with an average of 4 DP) (Raninen et al., 2011; Roberfroid, 2007). Due to β (2,1) linkage, ITF is resistant to digestion in the small intestine and persisted into the colon where it undergoes fermentation. It is estimated that the Europeans and the Americans consume an average of 3–11 g and 1.3–3.5 g of ITF daily, respectively (van Loo et al., 1995). In 2018, inulin was approved by the Food and Drug Administration (FDA) in USA as an added dietary fiber so as to improve the nutritional value of the manufactured food products (Administration, 2018).

The data collected from animal models have indicated nutritional benefit of ITF in controlling obesity, diabetes, and hyperlipidemia, but only a few studies have been conducted to examine its effects on the human health (Beylot, 2005; Le Bourgot et al., 2018). Moreover, the data of human studies are not consistent, and thus, ITF benefit on human health is inconclusive. Therefore, the primary objective of this work was to comprehensively review the recent research performed on human subjects and carry out a meta‐analysis evaluating the beneficial effect of ITF, if any, on humans. Parameters included in this analysis consist of changes to body weight, blood glucose, insulin, and lipid profile induced by ITF intake in human subjects. The secondary objective was to summarize the possible mechanisms underlying the beneficial effects of ITF intake observed in this study.

## 2 MATERIALS AND METHODS

### 2.1 Data sources and literature search

A comprehensive literature search for human intervention studies that evaluated the correlation between ITF intake and body weight, blood glucose or lipid profile, published between 1960 and 2020 was performed. NCBI, PubMed, Scopus, Ovid, EBSCO, Web of Science, ProQuest databases, Science, JSTOR, and MEDLINE were searched using the search terms ‘inulin‐type fructans’, OR ‘inulin’ OR ‘fructo‐oligosaccharides’ OR ‘oligofructose’ AND ‘blood glucose’ OR ‘lipid’ OR ‘cholesterol’ OR ‘triglycerides’ AND ‘human’, and the same terms were applied in each database during the search phase. In addition, a manual search of the reference lists of retrieved papers or review articles was conducted to identify all potentially relevant papers. No limit was placed on the language. Data were extracted by two reviewers. The present meta‐analysis has been registered with PROSPERO (CRD42018117715).

The studies included in this review met the following criteria: (a) ITF intake exposure; (b) human subjects (≥18 years of age); (c) randomized controlled trial (RCT); and (d) data including body weight, blood glucose, insulin, or lipid profile. A study was excluded if it was an observational study, an animal study, a review or meta‐analysis, a trial in the general population, a trial without relevant effect measures, or a non‐ITF supplementation trial.

### 2.2 Data extraction

The following data were extracted from each human study: lead author, year of publication, health status of subjects, number of subjects (female and male), BMI, age (year), number of subjects in the intervention and control groups, type of ITF consumed, dosage, duration, control, study design, and outcomes. The study duration in this review strictly referred to either the period of inulin intervention or the control diet rather than overall study period. For the missing data that were not explained in the corresponding articles, authors were contacted via email or phone call to seek permission for the data to be included. In cases where there is no answer, request refusal or data loss, the missing data were reported as “Not stated.”

The Heyland Methodologic Quality Score (MQS) was used to assess study quality (Heyland et al., 2001). Points were given on the basis of methodology (randomization, blinding, and analysis), sample (selection, compatibility, and follow‐up), and intervention (protocol, co‐intervention, and crossovers). A maximum of 13 points could be received. Study that received a score of 8 was considered to be of higher quality.

### 2.3 Statistical analysis

Reviewer Manager (RevMan, version 5.3) was used to conduct the meta‐analysis with changes in body weight, blood glucose, insulin, and lipid profile including total cholesterol, LDL cholesterol, HDL cholesterol, and triglycerides (TAG) as the outcomes. Heterogeneity across studies was quantified by using the *I* ^2^ statistic methodology, where each study design was considered, as a quantitative evaluation of inconsistency among the studies. To pool the results of studies with an acute impact on body weight, blood glucose, insulin, and lipid profile, a fixed‐effects model was selected when heterogeneity was absent or low (*I* ^2^ < 20%), whereas when heterogeneity was greater, a random‐effects model was utilized. In this work, weighted mean differences (WMDs) between treatment (ITF) and control groups were combined via a random‐effects model to evaluate the size of treatment impacts. When the *I* ^2^ value is ≥20%, in which case the source of heterogeneity was explored by the removal of individual trials in the sensitivity analyses and through a priori subgroup analyses.

To examine whether a single study exerted undue influence on the overall results, sensitivity analyses were performed in which each individual study was excluded from the meta‐analysis and the effect size was recalculated with the remaining studies. Meanwhile, a priori subgroup analyses were performed to further identify the possible sources of heterogeneity by comparing summary results obtained from subsets of studies grouped by characteristics (prediabetes or diabetes), study design, dosage, and duration. For this study, the potential publication bias was evaluated with STATA 12.0. Here, visual inspection of Funnel plots and quantitatively assessment using Begg's and Egger's tests were performed. A *p* < .05 was deemed statistically significant for all analyses in this study.

## 3 RESULTS

### 3.1 Literature search

**FIGURE 1:**
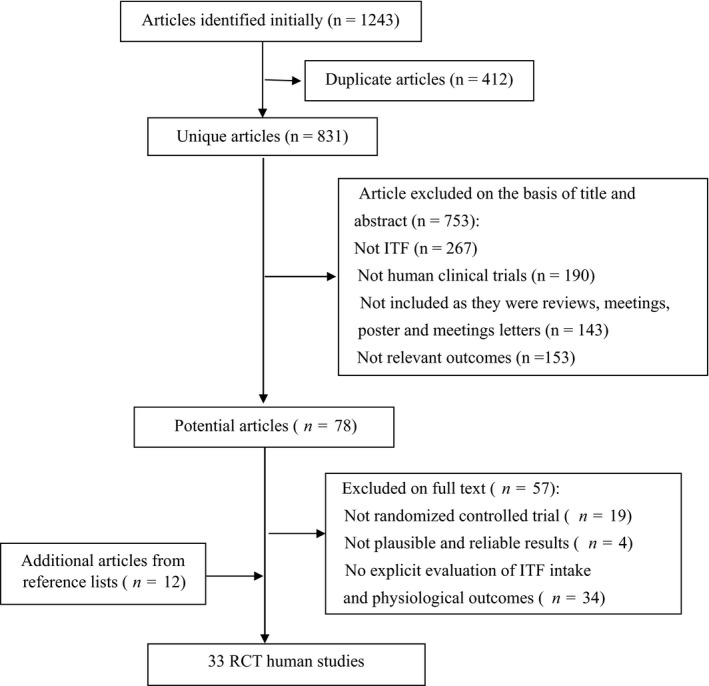
Flow diagram of article selection

As shown in Figure 1, 1,243 articles were identified and evaluated in the initial systematic search on the scientific databases. Upon the removal of the duplicate articles (412) and articles that did not meet the eligibility criteria (810), a total of 21 human studies were included in this review. It is noteworthy that three trials performed by Dehghan et al. (2013), Dehghan et al. (2014) and Bahram Pourghassem et al. (2013) evaluated the effects of inulin consumption (10 g/day for 8 weeks) in the same population (49 Iranian women with type 2 diabetes). Their data showed a 35.3% reduction in LDL cholesterol concentration, which is comparable to the effect of a statin drug (Deedwania et al., 2005). Recently, Dehghan et al. (2016) performed a study based on a similar population (46 Iranian women with type 2 diabetes) and demonstrated that the intake of FOS‐enriched inulin (10 g/day for 2 months) decreased LDL cholesterol concentration from 116.04 to 36.97 mg/dl. Interestingly, the effect far exceeded the effect of a high‐dose/high‐impact statin drug (Law et al., 2003). Taken together, due to their highly implausible and unreliable results, the above studies were excluded from this review to ensure the accuracy of this meta‐analysis. Manual searches performed on the reference lists of the relevant articles yielded 12 additional articles. Consequently, the combination of electronic and manual searches resulted in 33 articles which were included in the final review. Four human studies were conducted in UK; three in Iran, France, Spain, the Netherlands, and Belgium; two in USA, Canada, Mexico, and Japan; and one in China, Denmark, Italy, Brazil, Canada, and Argentina.

### 3.2 Study characteristics

Extracted data from the human studies are summarized in Table 1. All studies in this review were RCT studies, with 57% (19 trials) utilizing a parallel design, 40% (13 trials) utilizing a crossover design, and 3% (one trial) utilizing a Latin‐square design. The subjects included in this analysis were stratified into healthy or one of the following metabolic symptomatic groups, including overweight and obese, prediabetes and diabetes, and hyperlipidemia. Of the 33 trials, the number of studies that had utilized healthy, overweight and obese, prediabetes and diabetes, and hyperlipidemia subjects as study objects was 8, 10, 10 and 5, respectively. Overall, ITF intake in RCT human studies ranged from 3 to 30 g/day of ITF as part of the ingredient in the diet (11 g, median levels of individual series). ITF was provided as beverages, ice cream, or natural solid foods such as cookies, pasta, and bread rolls. Treatment duration ranged from 2 to 18 weeks with the median length of 6 weeks. Blood samples were obtained at baseline and after diet intervention. Other parameters including body weight, blood glucose, insulin, or lipid profile were collected.

**TABLE 1:** Summary of 33 human studies reviewed

Source	Subjects				Intervention			Control	Study design	MQS	Outcomes 95% CIs
	Health status	BMI	Age	*N* (E/C)	Type	Dosage	Duration				
Luo et al. (1996)	Healthy (12M)	21	24	12 (12/12)	FOS	20 g/day	4 weeks	Sucrose	Crossover RCT double‐blind	8	↔Body weight, FPG, insulin, TC, HDL‐C, TG, Apo A1, Apo B, lipoprotein (a); ↓Basal hepatic glucose production
Pedersen et al. (1997)	Healthy (64F)	21.9	20–36	64 (64/64)	Inulin	14 g/day	4 weeks	Control spread without inulin	Crossover RCT double‐blind	9	↔Energy intake, TC, LDL‐C, HDL‐C, TG, LDL‐C/HDL‐C
van Dokkum et al. (1999)	Healthy (12M)	Not stated	23	12 (12/12)	Inulin		3 weeks	Control diet without inulin	Latin square RCT double‐blind	7	↔Body weight, glucose tolerance test, TC, LDL‐C, HDL‐C, TG, Apo A1, Apo B, fecal neutral steroids; ↑Fecal acetate and valerate
Letexier et al. (2003)	Healthy (4F and 4M)	19–25	23–32	8 (8/8)	Inulin	10 g/day	3 weeks	Maltodextrin	Crossover RCT double‐blind	6	↔Body weight, FPG, insulin, glucagon, TC, LDL‐C, HDL‐C, NEFA; ↓ TG, hepatic lipogenesis
Forcheron and Beylot (2007)	Healthy (11F and 6M)	Not stated	32	17 (9/8)	Inulin + FOS (1:1)	10 g/day	6 weeks	Control without inulin	Parallel RCT double‐blind	6	↔Body weight, fat mass, FPG, insulin, glucagon, TC, LDL‐C, HDL‐C, TG, NEFA, cholesterol synthesis
Russo et al. (2010)	Healthy (22M)	22.8	18.8	22 (22/22)	Inulin	11 g/day	5 weeks	Control pasta without inulin	Crossover RCT double‐blind	10	↔Energy intake, insulin, TC, LDL‐C; ↓ FPG, HbA1c, TG, lipoprotein (a), HOMA‐IR; ↑ HDL‐C
Garcia‐Garcia et al. (2013)	Healthy (not stated)	25.1	Not stated	32 (17/15)	Inulin	1.5 g/day	4 weeks	Confection without inulin	Parallel RCT double‐blind	7	↔Body weight, glycosylated hemoglobin (%), TC, LDL‐C, HDL‐C, TG
Scheid et al. (2014)	Healthy (not stated)	27.9	67.1	72 (37/35)	FOS	7.4 g/day	9 weeks	Maltodextrin	Parallel RCT double‐blind	7	↔Insulin, TC, LDL‐C, VLDL‐C, HDL‐C, TG, CRP, HOMA‐IR; ↓ FPG
Parnell and Reimer (2009)	Overweight and obese (32F and 7M)	30.1	40.4	39 (21/18)	FOS	21 g/day	12 weeks	Maltodextrin	Parallel RCT double‐blind	9	↔Appetite, hunger, TC, LDL‐C, HDL‐C, TG; ↓ Energy intake, body weight, FPG; ↑ Insulin, active tAUC PYY
Genta et al. (2009)	Obese and mild dyslipidemic (35F)	34	41	35 (20/15)	FOS	10 g/day	17 weeks	Control syrup	Parallel RCT double‐blind	7	↔FPG, TC, HDL‐C, TG; ↓ Body weight, BMI, insulin, LDL‐C, HOMA‐IR
de Luis et al. (2010)	Obese (26F and 4M)	37.9	56.0	30 (15/15)	Inulin	3 g/day	4 weeks	Control cookies without inulin	Parallel RCT double‐blind	11	↔Body weight, BMI, FPG, insulin, HDL‐C, TG, HOMA‐IR, CRP, QUICKI; ↓ TC, LDL‐C
de Luis et al. (2013) (117)	Obese (27F and 9M)	37.6	25–60	36 (18/18)	FOS	9.84 g/day	4 weeks	Control cookies without inulin	Parallel RCT double‐blind	10	↔Body weight, BMI, fat mass, FPG, insulin, TC, LDL‐C, HDL‐C, TG, CRP, HOMA‐IR; ↑ Satiety
Tripkovic et al. (2015)	Obese (10M)	30.2	39.8	10 (10/10)	Inulin	15 g/day	4 weeks	Refined wheat grain	Crossover RCT double‐blind	7	↔Body weight, BMI, body fat percentage, FPG, insulin, TC, HDL‐C, TG, NEFA, HOMA‐IR
Tovar et al. (2012)	Overweight and Obese (59F)	30.8	33.0	59 (30/29)	Inulin	10 g/day	12 weeks	Control without inulin	Parallel RCT not‐blind	11	↔Body weight, BMI, FPG, TC, LDL‐C, HDL‐C; ↓ TG
Dewulf et al. (2013)	Obese (30F)	35.9	47.5	30 (15/15)	Inulin + FOS (1:1)	16 g/day	12 weeks	Maltodextrin	Parallel RCT double‐blind	10	↔BMI, waist/hip ratio, fat mass, FPG, insulin, TC, LDL‐C, HDL‐C, TG, HOMA index, CRP
Daud et al. (2014)	Overweight and obese (16F and 6M)	30.3	33.0	22 (12/10)	FOS	30 g/day	6 weeks	Maltodextrin + cellulose	Parallel RCT single‐blind	8	↔Body weight, BMI, FPG, insulin, TC, LDL‐C, HDL‐C, TG, HOMA‐IR, PYY, GLP‐1, AST, ALT, tAUC glucose, tAUC insulin; ↓ tAUC hunger and motivation to eat; ↑ tAUC PYY
Castro‐Sanchez et al. (2016)	Obese and dyslipidemic (not stated)	35.9	Not stated	16 (16/16)	Inulin	9 g/day	8 weeks	Dextrose	Crossover RCT double‐blind	7	↔Body weight, BMI, body fat percentage, TC, LDL‐C, TG; ↑ HDL‐C
Pol et al. (2018)	Overweight or obesity (36F and 19M)	29.7	40.6	55 (29/26)	FOS	16 g/day	12 weeks	Control without FOS	Parallel RCT triple‐blind	11	↔Energy intake, satiety, appetite, body weight, body composition
Yamashita et al. (1984)	Type 2 diabetes (not stated)	Not stated	Not stated	28 (18/10)	FOS	8 g/day	2 weeks	sucrose	Parallel RCT double‐blind	8	↔HDL‐C, TG, NEFA; ↓ FPG, TC, LDL‐C
Luo et al. (2000)	Type 2 diabetes (4F and 6M)	28	57	10 (10/10)	FOS	20 g/day	4 weeks	Sucrose	Crossover RCT double‐blind	6	↔Body weight, FPG, insulin, HbA1c (%), TC, LDL‐C, HDL‐C, TG, NEFA, Apo A1, Apo B, lipoprotein (a), basal hepatic glucose production
Alles et al. (1999)	Type 2 diabetes (11F and 9M)	28.3	59.3	20 (20/20)	FOS	15 g/day	3 weeks	Glucose	Crossover RCT double‐blind	7	↔Body weight, FPG, TC, LDL‐C, HDL‐C, TG, NEFA
Bonsu and Johnson (2012)	Type 2 diabetes (12F and 14M)	30.3	65.1	26 (12/14)	Inulin	10 g/day	12 weeks	Xylitol	Parallel RCT double‐blind	6	↔FPG, HbA1c (%), TC, LDL‐C, HDL‐C, TG, TC/HDL‐C
Guess et al. (2014)	Prediabetes (not stated)	Not stated	Not stated	33 (33/32)	Inulin	30 g/day	2 weeks	Cellulose	Crossover RCT double‐blind	5	↔ FPG, insulin, HOMA‐IR; ↓ Body weight
Kellow et al. (2014)	Prediabetes (21F and 6M)	33	52.3	27 (27/27)	Inulin + FOS	10 g/day	12 weeks	Maltodextrin	Crossover RCT double‐blind	9	↔Body weight, BMI, FPG, insulin, TC, LDL‐C, HDL‐C, TG, HOMA‐IR, high‐sensitivity CRP, ALT; ↑ HDL‐C
Liu et al. (2015)	Type 2 diabetes (14F and 36M)	Not Stated	63.5	50 (25/25)	Inulin	15 g/day	8 weeks	Control without inulin	Parallel RCT	7	↔HDL, TG, AST, ALT; ↓FPG, HbA1c, TC, LDL‐C, HOMA‐IR
Aliasgharzadeh et al. (2015)	Type 2 diabetes (52F)	30.9	48.4	52 (27/25)	Inulin	15 g/day	8 weeks	Maltodextrin	Parallel RCT triple‐blind	7	↔Energy intake, HDL‐C, TG; ↓ Body weight, BMI, FPG, HbA1c, TC, LDL‐C
Roshanravan et al. (2017)	Type 2 diabetes (17F and 13M)	30.6	51.6	30 (15/15)	Inulin	10 g/day	6 weeks	Starch powder	Parallel RCT double‐blind	10	↔Body weight, BMI, FPG, insulin, HbA1c, HOMA‐IR, GLP‐1, TC, LDL‐C, HDL‐C, TG; ↑ GLP‐1
Ghavami et al. (2018)	Type 2 diabetes (26F and 20M)	28.3	42.1	46 (23/23)	Inulin	10 g/day	6 weeks	Starch powder	Parallel RCT double‐blind	11	↔Body weight, BMI, insulin, HbA1c (%), TC, LDL‐C, HDL‐C, TG, HOMA‐IR; ↓ FPG
Hidaka et al. (1991)	Hyperlipidemic (F and M)	Not stated	Not stated	37 (20/17)	FOS	8 g/day	5 weeks	Sucrose	Parallel RCT double‐blind	6	↔Body weight, FPG, HDL‐C, TG, NEFA; ↓ TC
Davidson et al. (1998)	Hypercholesterolemic (F and M)	Not stated	30–75	21 (21/21)	Inulin	18 g/day	6 weeks	Control food without inulin	Crossover RCT double‐blind	5	↔Body weight, HDL‐C, LDL‐C/HDL‐C, TG; ↓ TC, LDL‐C
Jackson et al. (1999)	Moderately increased TC and TG (F and M)	26.3	52.3	54 (27/27)	Inulin	10 g/day	8 weeks	Maltodextrin	Parallel RCT double‐blind	9	↔Weight body, FPG, insulin, TC, LDL‐C, HDL‐C, Apo A1, Apo B; ↓ TG
Causey et al. (2000)	Mild hypercholesterolemia (12 M)	Not stated	27–49	12 (12/12)	Inulin	20 g/day	3 weeks	Control ice cream without sucrose	Crossover RCT double‐blind	7	↔TC, LDL‐C, HDL‐C, Apo A1, Apo B, SCFA, fecal total bile acids; ↓ TG
Giacco et al. (2004)	Mild hypercholesterolemia (10F and 20M)	26.6	45.5	30 (30/30)	FOS	10.6 g/day	8 weeks	Maltodextrin and cellulose	Crossover RCT double‐blind	8	↔Body weight, FPG, insulin, TC, LDL‐C, HDL‐C, TG, NEFA, Apo A1, Apo (a), postprandial glucose; ↑ Postprandial insulin

Abbreviations: ALT, aspartate aminotransferase; Apo A1, apolipoprotein A1; Apo B, apolipoprotein B; AST, alanine aminotransferase; CRP, C‐reactive protein; FOS, fructo‐oligosaccharides; FPG, fasting plasma glucose; GLP‐1, glycogen like peptide 1; HbA1c, hemoglobin a1c; HDL‐C, high‐density lipoprotein‐cholesterol; HOMA‐IR, homeostasis model assessment‐insulin resistance; LDL‐C, low‐density lipoprotein‐cholesterol; MQS, Methodologic Quality Score; NEFA, nonesterified fatty acid; PYY, peptide YY; QUICKI, quantitative insulin sensitivity check index; RCT, randomized controlled trial; SCFA, short‐chain fatty acids; TAG, triglycerides; TC, total cholesterol.

### 3.3 Effect on body weight

**FIGURE 2:**
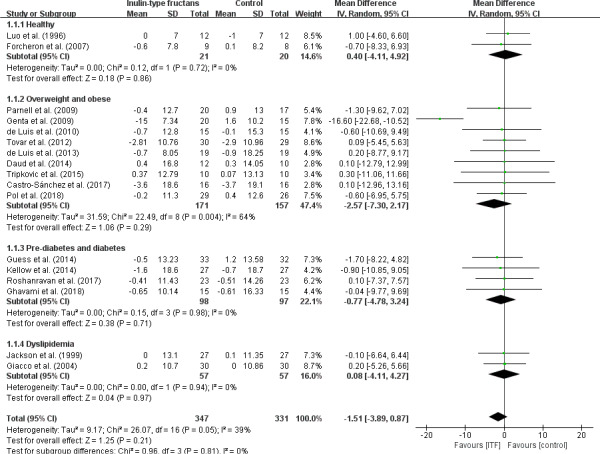
Forest plot of randomized controlled trials investigating the effect of ITF intake on body weight (kg) compared with control groups for human studies. Results from individual trials were pooled with random‐effects model and are expressed as weighted (squares) mean differences with 95% CIs (horizontal lines). Pooled effect estimates (diamonds) are presented for each subgroup as well as the overall analysis

To assess the intake of ITF in assisting with weight loss, a total of 17 studies were included in this analysis (Figure 2). The result showed that ITF intake had no effect on the body weight in the overall analysis (WMD, −1.51 kg; 95% CI: −3.89, 0.87; *p* = .21) and any subgroups (*p* > .05). There was a significant intertrial heterogeneity in the overweight and obese subgroup (*I* ^2^ = 64%, *p* = .004), as well as borderline significance in intertrial heterogeneity in the overall pooled analysis (*I* ^2^ = 39%, *p* = .05).

### 3.4 Effect on blood glucose

**FIGURE 3:**
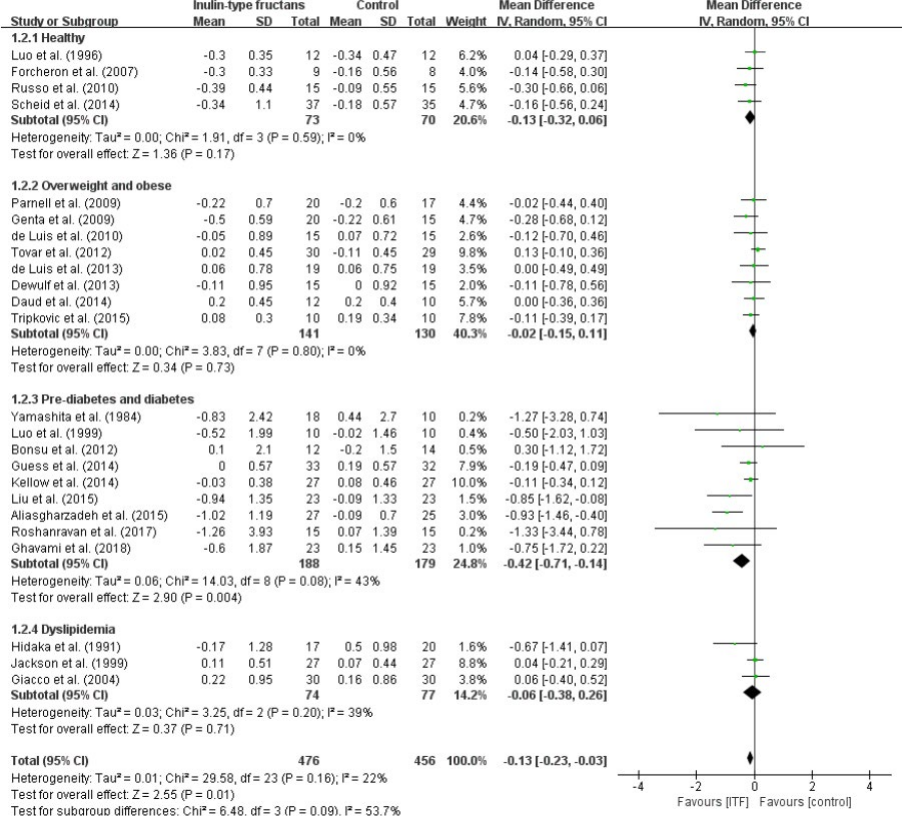
Forest plot of randomized controlled trials investigating the effect of ITF intake on blood glucose (mmol/L) compared with control groups for human studies. Results from individual trials were pooled with random‐effects model and are expressed as weighted (squares) mean differences with 95% CIs (horizontal lines). Pooled effect estimates (diamonds) are presented for each subgroup as well as the overall analysis

Results for blood glucose were reported in 24 eligible studies (Figure 3). Overall, ITF led to lower glucose concentration when compared to a control diet (WMD, −0.13 mmol/L; 95% CI: −0.23, −0.03; *p* = .01). The studies on prediabetes and diabetes subjects accounted for 24.8% of the weight in the analysis; in the stratified analysis, these studies alone suggested a positive effect of greater magnitude on patients with prediabetes and diabetes compared with total subjects (WMD, −0.42 mmol/L; 95% CI: −0.71, −0.14; *p* = .004). The overall test for heterogeneity resulted in *I* ^2^ = 22% (*p* = .16); when studies on patients with prediabetes and diabetes were removed from the analysis, heterogeneity was reduced (*I* ^2^ = 0%, *p* = .76). These suggested that studies on prediabetes and diabetes patients exert a large effect on the overall result.

### 3.5 Effects on insulin

**FIGURE 4:**
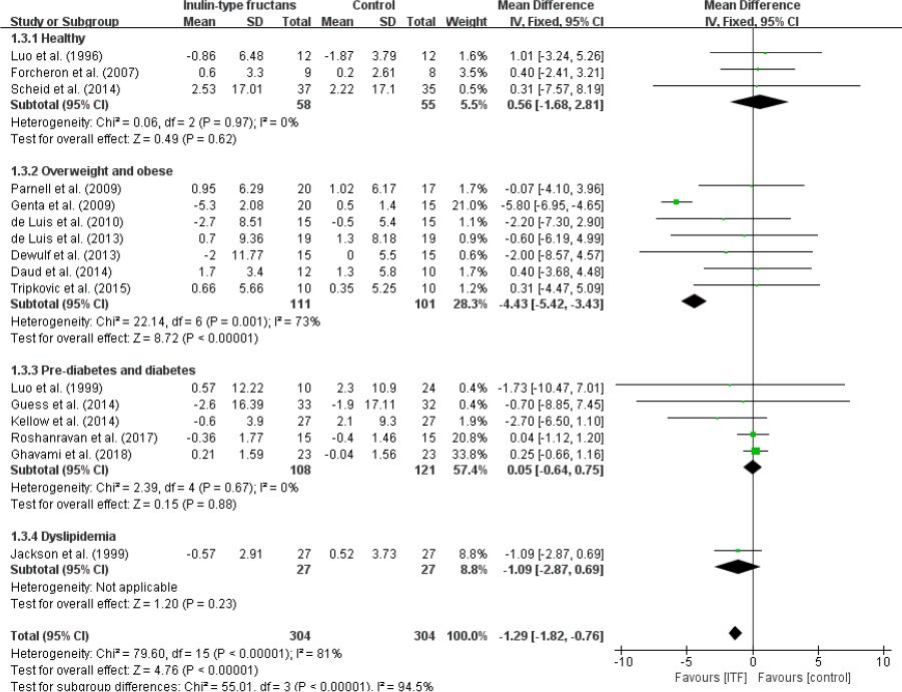
Forest plot of randomized controlled trials investigating the effect of ITF intake on plasma or serum insulin (µlU/ml) compared with control groups for human studies. Results from individual trials were pooled with random‐effects model and are expressed as weighted (squares) mean differences with 95% CIs (horizontal lines). Pooled effect estimates (diamonds) are presented for each subgroup as well as the overall analysis

Overall, significant reduction in insulin concentration was observed (WMD, −1.29 µlU/ml; 95% CI: −1.82, −0.76; *p* < .00001) when 16 trials were analyzed (Figure 4). The overall test for heterogeneity resulted in *I* ^2^ = 81% (*p* < .00001). With the exception of the overweight and obese subgroup (*I* ^2^ = 73%, *p* = .001), intake of ITF did not affect insulin concentrations in any other subgroups with no evidence of intertrial heterogeneity. The overall intertrial heterogeneity was substantially reduced to *I* ^2^ = 0% (*p* = .846) after the removal of the overweight and obese subgroup. Thus, this subgroup appeared to be the main source of heterogeneity.

### 3.6 Effects on total cholesterol

**FIGURE 5:**
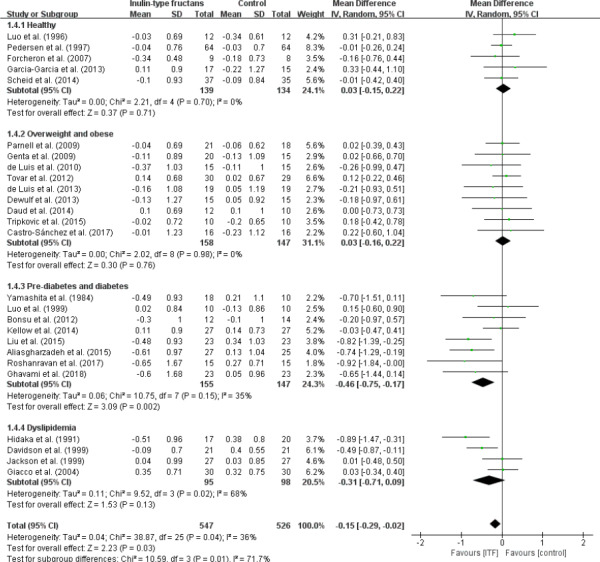
Forest plot of randomized controlled trials investigating the effect of ITF intake on total cholesterol (mmol/L) compared with control groups for human studies. Results from individual trials were pooled with random‐effects model and are expressed as weighted (squares) mean differences with 95% CIs (horizontal lines). Pooled effect estimates (diamonds) are presented for each subgroup as well as the overall analysis

Overall, a significant reduction in total cholesterol (WMD, −0.15 mmol/L; 95% CI: −0.29, −0.02; *p* = .03) was observed in 26 trials (Figure 5). The stratified analysis found that only prediabetes and diabetes subjects showed a significant reduction in total cholesterol concentration after ITF‐supplemented diet (WMD, −0.46 mmol/L; 95% CI: −0.75, −0.17; *p* = .002). This is based on a total of 8 trials, which accounted for almost one quarter of weight (24.3%) of the overall analysis. The overall test for heterogeneity resulted in *I* ^2^ = 36% (*p* = .04). Heterogeneity for total cholesterol was markedly reduced when studies on prediabetes and diabetes subjects were removed from the analysis (*I* ^2^ = 11.8%, *p* = .31). The data indicated that this group was largely responsible for the observed heterogeneity.

### 3.7 Effect on LDL cholesterol

**FIGURE 6:**
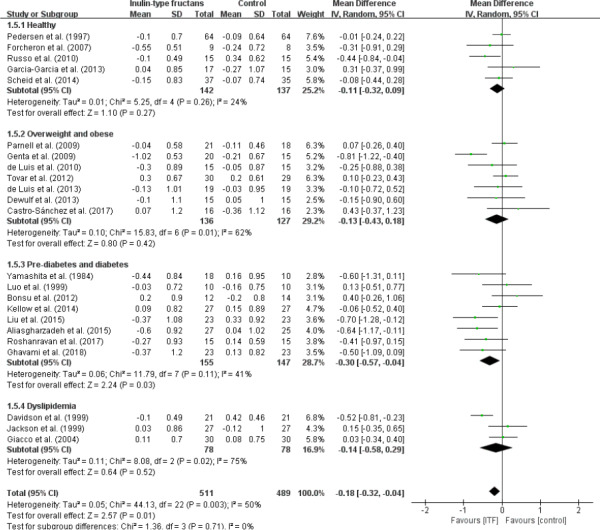
Forest plot of randomized controlled trials investigating the effect of ITF intake on LDL cholesterol (mmol/L) compared with control groups for human studies. Results from individual trials were pooled with random‐effects model and are expressed as weighted (squares) mean differences with 95% CIs (horizontal lines). Pooled effect estimates (diamonds) are presented for each subgroup as well as the overall analysis

In the 23 studies that evaluated LDL cholesterol, there was evidence of significant lowered LDL cholesterol concentrations in group with ITF intake (WMD, −0.18 mmol/L; 95% CI: −0.32, −0.04; *p* = .01; Figure 6). However, the stratified analysis showed that only prediabetes and diabetes LDL cholesterol concentrations were significantly decreased (WMD, −0.30 mmol/L; 95% CI: −0.57, −0.04; *p* = .03). Here, evidence of heterogeneity was present in all subgroups. In addition, *I* ^2^ was >20% in the overall analysis. The heterogeneity remained unchanged even with the removal of the prediabetes and diabetes groups (*I* ^2^ = 53.7%; *p* = .007).

### 3.8 Effect on HDL cholesterol

**FIGURE 7:**
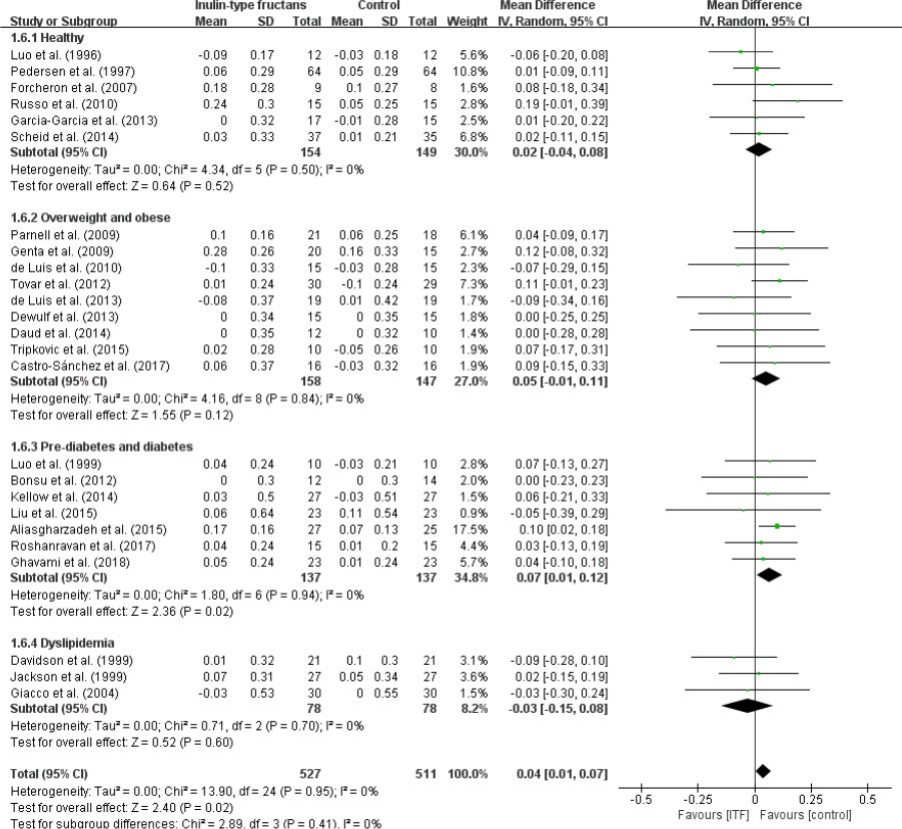
Forest plot of randomized controlled trials investigating the effect of ITF intake on HDL cholesterol (mmol/L) compared with control groups for human studies. Results from individual trials were pooled with random‐effects model and are expressed as weighted (squares) mean differences with 95% CIs (horizontal lines). Pooled effect estimates (diamonds) are presented for each subgroup as well as the overall analysis

Across the 25 studies’ analysis, intake of ITF significantly increased the concentration of HDL cholesterol in comparison with control group (WMD, 0.04 mmol/L; 95% CI: 0.01, 0.07; *p* = .02; Figure 7). The stratified analysis found that only studies performed on patients with prediabetes and diabetes, which accounted for almost one third of the weight (34.8%), showed reduction in HDL cholesterol concentrations (WMD, 0.07 mmol/L; 95% CI: 0.01, 0.12; *p* = .02; Figure 7). No evidence of heterogeneity in the overall analysis and all subgroups was detected with *I* ^2^ = 0% (*p* > .05).

### 3.9 Effect on triglyceride

**FIGURE 8:**
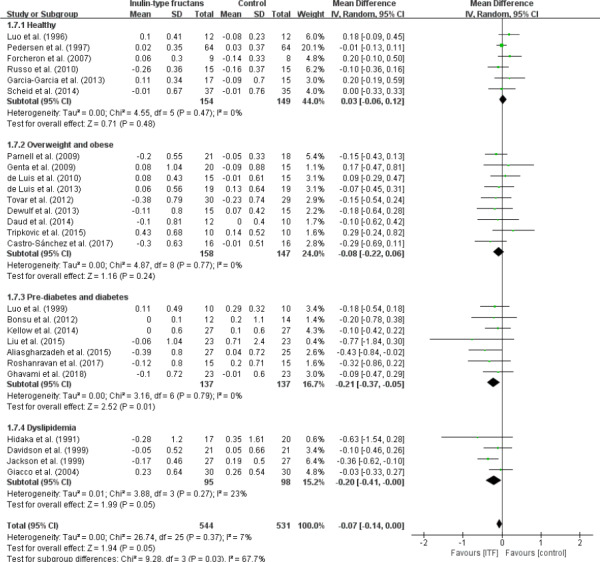
Forest plot of randomized controlled trials investigating the effect of ITF intake on triglycerides (mmol/L) compared with control groups for human studies. Results from individual trials were pooled with random‐effects model and are expressed as weighted (squares) mean differences with 95% CIs (horizontal lines). Pooled effect estimates (diamonds) are presented for each subgroup as well as the overall analysis

In this investigation, the borderline significance (WMD, −0.07 mmol/L; 95% CI: −0.14, 0.00; *p* = .05) effect of ITF intake on TAG was observed between ITF and control group as showed in a total of 26 studies, with little heterogeneity (*I* ^2^ = 7%, *p* = .37; Figure 8). Similarly, the effect among subjects with hyperlipidemia (WMD, −0.20 mmol/L; 95% CI: −0.41, 0.00; *p* = .05) was also marginally significant. As for the subgroup of subjects with prediabetes and diabetes, the concentration of TAG appeared to be significantly decreased (WMD, −0.21 mmol/L; 95% CI: −0.37, −0.05; *p* = .01) with seven trials.

### 3.10 Publication bias

Next, publication bias of these trials was examined by funnel plot analysis, Begg's test, and Egger's test (Figure S1). With exception to body weight and blood glucose, visual inspection of the funnel plots found that individual studies of WMD estimates were reasonably symmetrical. The absence of publication bias (*p* > .05) was supported by both Begg's test and Egger's test. For body weight, visual inspection of funnel plot indicated potential asymmetry, this was, however, not confirmed by Begg's test (*p* = .149) and Egger's test (*p* = .578). For trials that report blood glucose, statistical evidence of publication bias (Begg's test, *p* = .018; Egger's test, *p* = .006) was observed.

### 3.11 Side effects

Detailed information gathered from the 33 RCTs in this review showed that ITF was mostly well tolerated by subjects of different health status. However, minor side effects such as abdominal flatulence, bloating, and nausea were reported (Daud et al., 2014; de Luis et al., 2013; Guess et al., 2014). None of these effects were recognized as adverse to the human health, and the symptoms eventually subsided with diet adaptation over time (Parnell & Reimer, 2009). Nonetheless, it has been reported that adding 20 g FOS/70 kg body weight/day led to significant gastrointestinal side effects, which is not seen if 10 g/day was offered (Genta et al., 2009). Scientifically, from the view of gut microbiota modulation, slight flatulence and bloating are probably positive cues that the prebiotics is effective. Fermentation of prebiotics by the gut microbiota in the colon gave rise to the production of gas and acid, resulting in the symptoms.

## 4 DISCUSSION

ITF supplementation in diet has been suggested to alleviate several features of metabolic syndrome including obesity, diabetes, and hyperlipidemia; however, results from human trials remained inconsistent. This current study demonstrates that the favorable outcome of ITF intake was observed only in prediabetes and diabetes subjects, and favorable outcome was defined as significant blood glucose, total cholesterol, and TAG concentration reduction after study duration. Current data indicate that ITF only benefits individual of certain health status. Also, the removal of subjects with prediabetes did not change the significance of effects on blood glucose and lipid profiles; instead, increased significance was observed (data not shown).

Our current results are, in part, in line with a recent meta‐analysis where dietary prebiotic intake lowered the plasma TC, LDL‐C, and TAG concentrations of the diabetes trials included in their analysis (Beserra et al., 2015). The magnitude of the treatment response varies depending on the pathological state and the gut microbiota composition (Beserra et al., 2015; Larsen et al., 2010; Wu et al., 2010). Notably, a previous meta‐analysis by Liu et al. (2017) showed that ITF intake is beneficial by sustaining glucose homeostasis and reducing LDL‐C level; yet, there are several differences between Liu's meta‐analysis and ours. Firstly, in Liu's meta‐analysis, both studies by Dehghan et al. (2013) and Dehghan et al. (2014) (details of these two studies shown in Literature Search) were included, whereas considering the reliability of the dataset, they are excluded in our meta‐analysis. Secondly, in Liu's study, the analysis only considered the final data point at the end of treatment with no references to the baseline data point between the treatment groups. Therefore, the reported results could be misleading, since each treatment group has unique blood glucose and lipid value at baseline. In 2017, readers questioned the reliability of Liu's results based on the above facts and they also voiced their disagreement to the authors’ conclusions (Mcrorie et al., 2017). The above reasons prompted us to conduct a new systematic review and meta‐analysis. Here, our studies excluded Dehghan's studies and increase the number of RCT from 20 to 33. Furthermore, the effect of ITF intake on body weight which was absent in Liu's study was also carefully examined in our study.

### 4.1 Effect on body weight

There are increasing epidemiological studies, suggesting that dietary‐fiber‐rich diets are linked to lower body weight or BMI (Du et al., 2010; Grube et al., 2013; Thompson et al., 2017). Evidence from several animal studies has shown that ITF supplementation lowers energy intake through diet, promotes weight loss, and improves body composition like reduction in fat mass (Arora et al., 2012; Cani et al., 2004; Cani, Neyrinck, et al., 2005; Delmée et al., 2006; Dewulf et al., 2011). Above studies have speculated that fermentation of the ITF in the gut resulted in the higher concentration of peptide YY (PYY) and glucagon‐like peptide 1 (GLP‐1) via the short‐chain fatty acids (SCFA). Consistently, independent of other lifestyle changes, a 12‐week treatment with 21 g/day FOS in subjects who are overweight and obese has been shown to decrease energy intake through diet and body weight. This is likely due to suppressed ghrelin and enhanced PYY, but not GLP‐1, which remained unchanged (Parnell & Reimer, 2009). However, based on the results from this meta‐analysis, it appeared that the beneficial effects of ITF intake were not associated with weight loss. This is probably due to the fact that most studies did not include restricted energy intake through diet or intention to lose weight as one of the factors in their data analysis.

### 4.2 Effect on glucose

The current meta‐analysis showed that ITF intake significantly decreased blood glucose concentration in prediabetes and diabetes subjects. Evidently, this beneficial effect was also supported by several diabetes animal model studies (Bharti et al., 2013; Byung‐Sung, 2011; Cani, Daubioul, et al., 2005; Cani et al., 2006; Gobinath et al., 2010; Ning et al., 2017; Zhang et al., 2018). Due to the lack of viscosity property, ITF is not associated with relatively low absorption in the gut. Despite this, there are several mechanisms that may be responsible for the effect of ITF on blood glucose. It is generally recognized that ITF, via the fermentation by the intestinal microbiota, produces high level of SCFA end products. Of which, 90%–95% comprised of acetate, butyrate, and propionate. Butyrate directly increases the PYY/proglucagon (the gene that encodes GLP‐1) gene expression both in vitro and in vivo in rat study (Zhou et al., 2006). Accordingly, it had been reported that FOS significantly promotes the expression and secretion of colonic GLP‐1 amide (7–36), and the antidiabetic impact of FOS is dependent on the action of GLP‐1 in the streptozotocin‐induced diabetic rats (Cani, Daubioul, et al., 2005; Cani et al., 2006). Consistently, Zhao et al. (2018) also found that increased fecal butyrate concentrations coincided with a significantly greater postprandial GLP‐1 area under the curve in diabetics consuming high‐fiber diets compared with the control. Acetate and propionate absorbed into the bloodstream were taken up by various peripheral tissues and organs such as liver, where they play an important role in various physiological processes. SCFA propionate is converted into methylmalonyl‐CoA and succinyl‐CoA, which in turn inhibit pyruvate carboxylase and gluconeogenesis (dos Reis et al., 2015). One other mechanism further suggested that ITF indirectly enhanced glycolysis. Here, propionate depletes hepatic citrate, which is the main inhibitor of one of the most important regulatory enzymes of glycolysis‐phosphofructokinase (Roberfroid & Delzenne, 1998).

### 4.3 Effect on cholesterol

In the medical field, treatments aimed to decrease cholesterol concentration are effective ways of combating the risk of cardiovascular diseases (Lloyd‐Jones et al., 2010; Yusuf et al., 2004). The cholesterol‐lowering effects of ITF observed may be accounted for by several mechanisms. Firstly, ITF mediates in vivo bile acid level. In inulin‐fed rats, lower serum cholesterol concentration was accompanied by an increase in fecal bile acid and neutral steroid excretion (Han et al., 2013; Levrat et al., 1994; Parnell & Reimer, 2010). The loss of bile acids in the feces enhanced cholesterol uptake by the liver from the circulation to replenish the bile acid supply. It has been reported that fecal bile acid excretion is inversely correlated with liver cholesterol concentrations (*r* ^2^ = .20) (Vanhoof & De Schrijver, 1995). Consistently, Yang and colleagues further suggested that the suppressive effect of ITF on cholesterol was mediated by the inhibition of cholesterol de novo synthesis as evidenced by the reduced *Srebf2* and *Hmgcr* gene expression. Due to its low viscosity, ITF does not bind to the bile acids in the intestinal lumen (Schneeman, 1999). But intestinal pH is lowered as the result of the organic acids produced during ITF fermentation in the intestinal mucosa. Consequently, the bile acids become less soluble and excreted with the feces, thereby reducing their intestinal absorption (Pereira & Costa, 2002). Secondly, as discussed previously, both acetate and propionate enter the liver through the portal vein. In rat administrated with FOS, both acetate and propionate concentrations risen more than twofold in the portal serum of the rats (Roberfroid & Delzenne, 1998). However, the role of SCFA in hypocholesterolemic action is difficult to verify. This is due to their antagonistic property: Acetate is a lipogenic substrate participating in the cholesterol biosynthesis and lipogenesis, while propionate prevents acetate uptake and inhibits fatty acid synthesis in the isolated hepatocytes of normal and Zucker rats (Daubioul et al., 2002; Demigne et al., 1995; Lin et al., 1995; Nishina & Freedland, 1990). The latter is supported by Daubioul et al study where they reported that 0.3 and 0.6 mmol/L propionate are able to reduce the incorporation of acetate into total lipids by 30% in the cultured isolated hepatocytes from obese Zucker rats, thereby depressing the lipogenesis process (Daubioul et al., 2002).

### 4.4 Effect on triglyceride

In rats, mice, and hamsters fed with ITF‐supplemented diet, hepatic lipid metabolism was regulated with reduced TAG accumulation in the hepatic and/or reduced serum lipids. Several mechanisms were proposed. Firstly, studies in the animal models clearly showed that lowered hepatic and/or serum TAG concentration were mainly due to the inhibition of TAG‐rich VLDL particle secretion (Delzenne et al., 2007; Kok, Roberfroid, & Delzenne 1996). It has been proposed that the hypotriglyceridemic action is probably attributed to the downregulation of de novo fatty acid synthesis in the liver, as evidenced by 50% decrease in the key hepatic lipogenesis enzyme activities, such as acetyl‐CoA carboxylase, fatty acid synthase, and malic enzyme. This is also reflected by a substantial reduction in fatty acid synthase mRNA, supporting the hypothesis that FOS treatment can modify lipogenic enzyme gene expression (Daubioul et al., 2002; Delzenne & Kok, 2001). Secondly, there were evidences suggesting that ITF may prevent the esterification step, in which hepatocytes isolated from FOS‐fed rats showed decreased capacity to esterify ^14^C‐palmitate and ^14^C‐acetate into TAG by 40% and 54%, respectively (Fiordaliso et al., 1995; Kok, Roberfroid, Robert, et al., 1996). However, the high levels of fat present in most human diets mean that the rate of de novo fatty acid synthesis in the liver is extremely low, as the exogenous dietary fatty acids provided all the required substrates for triglyceride VLDL synthesis. Thus, the explanation above cannot be extrapolated to humans (Aarsland et al., 1996). Thirdly, there is a possibility that ITF reduces serum TAG through an extrahepatic mechanism, namely through triglyceride‐rich lipoprotein catabolism enhancement (Daubioul et al., 2000; Delzenne et al., 2002). In one study, *ob/ob* rat fed with FOS displayed lower plasma TAG and muscle lipids. The phenomenon can be ascribed to 70% increase in the lipoprotein lipase mRNA expression in the muscle tissue (Everard et al., 2011).

### 4.5 Limitations

No study is without limitations. Firstly, the limited number of studies conducted to date, combined with small sample sizes and short intervention periods, insufficiently powered to support the effect, thus limiting the generalizability of observed effects on blood glucose and lipid profiles to larger populations with ITF intake. Secondly, there was considerable variability in study design between studies within subgroup of prediabetes and diabetes included in the meta‐analyses, with different study durations and dose of ITF consumed. These effects should be taken into account in meta‐regression analysis, but testing the number of studies available for such analyses is still inadequate. Finally, gap remained to fully elucidate the mechanisms underlying the protective effects on blood glucose and lipid profiles in subjects with prediabetes and diabetes.

## 5 CONCLUSION

In conclusion, the present meta‐analysis proposed that increased intake of ITF significantly reduces blood glucose, total cholesterol, and TAG in subjects with prediabetes and diabetes, without affecting the other subject groups. Because of the results of our study and the findings from previous studies, patients with prediabetes and diabetes may benefit from inulin supplementation in the reduction in blood glucose and the amelioration of the underlying health conditions. Several mechanisms were proposed to explain each health benefit that occurred in patients who are prediabetes and diabetes, but they remain inconclusive. Further research, especially large, well‐powered, long‐term human intervention studies, is required to further understand and promote the role that ITF plays in human health management.

## CONFLICT OF INTEREST

There are no conflicts of interest to declare.

## AUTHOR CONTRIBUTIONS

**Liangkui Li:** Formal analysis (lead); Methodology (equal); Resources (lead); Software (lead); Writing‐original draft (lead). **Peng Li:** Conceptualization (lead); Methodology (lead); Project administration (lead); Writing‐review & editing (lead). **Li Xu:** Conceptualization (lead); Data curation (lead); Methodology (lead); Project administration (lead); Writing‐review & editing (lead).

## Supporting information

Figure S1

## Data Availability

The data that support the findings of this study are available on request from the corresponding author.
