# Supplementary figures and images for: Assessing the effects of inulin‐type fructan intake on body weight, blood glucose, and lipid profile: A systematic review and meta‐analysis of randomized controlled trials
Source: Food Sci Nutr. 2021 Jun 21;9(8):4598–616. doi: 10.1002/fsn3.2403 (PMC8358370; doi:10.1002/fsn3.2403)

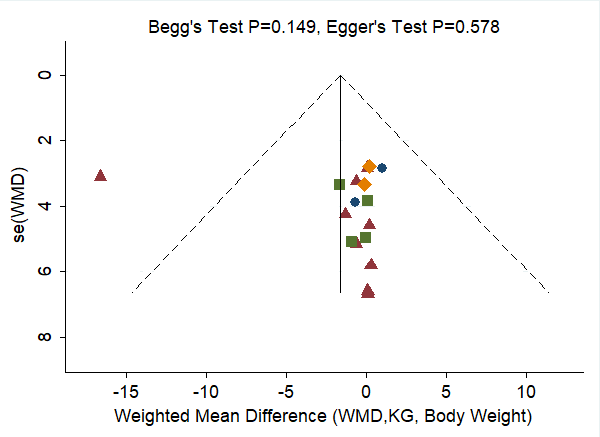

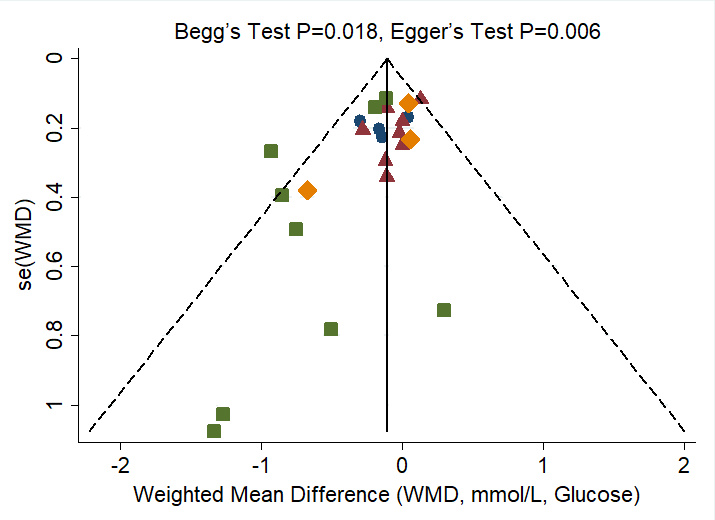


B

A

C


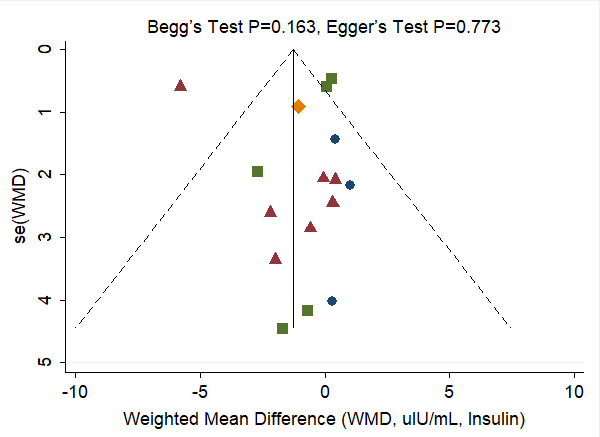

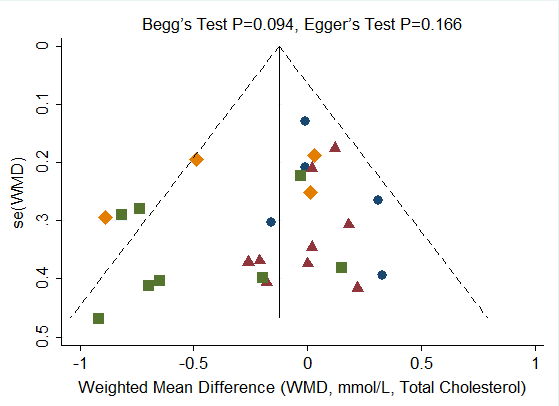


D


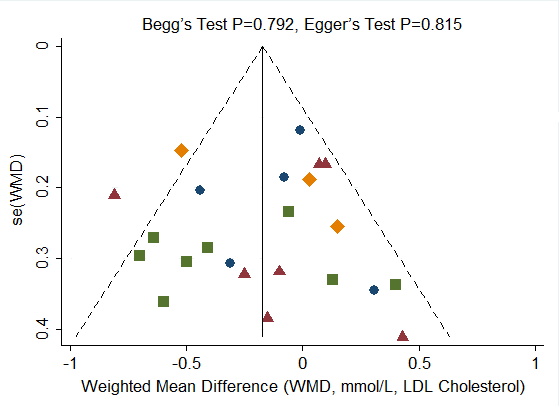

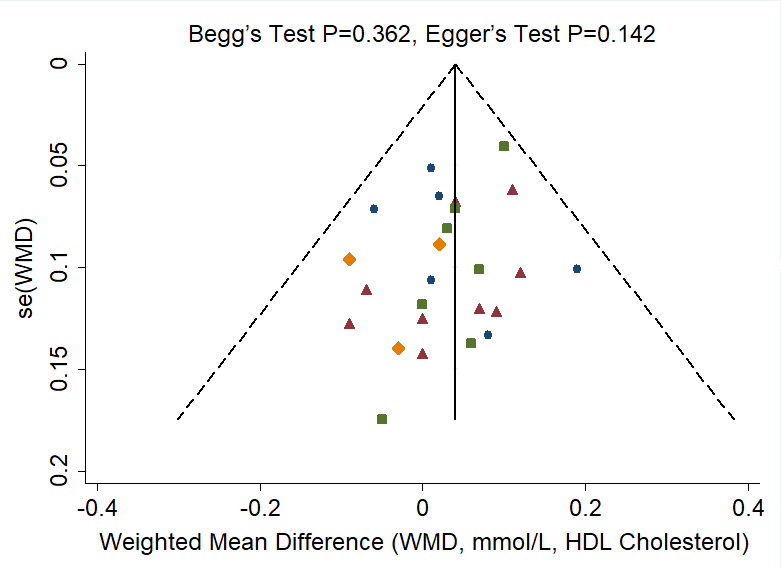


G

F

E


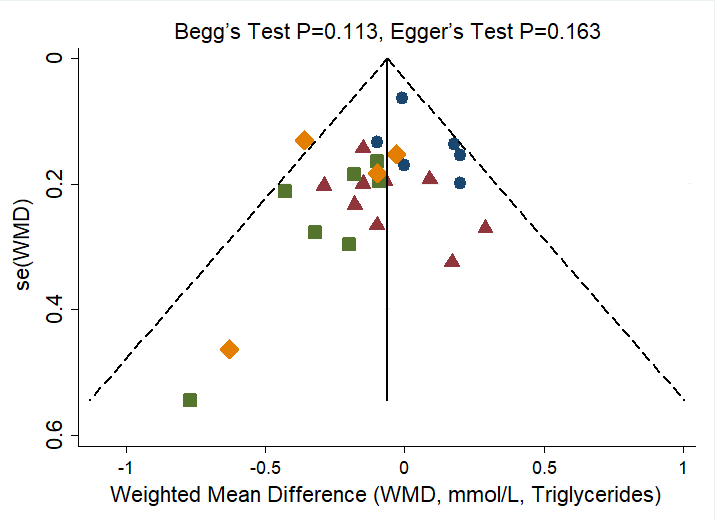

Supplement: Supplementary file 1 — Figure S1 [file FSN3-9-4598-s001.docx]
